# Lipid accumulation, lipid oxidation, and low plasma levels of acquired antibodies against oxidized lipids associate with degeneration and rupture of the intracranial aneurysm wall

**DOI:** 10.1186/2051-5960-1-71

**Published:** 2013-10-28

**Authors:** Juhana Frösen, Riikka Tulamo, Tommi Heikura, Sini Sammalkorpi, Mika Niemelä, Juha Hernesniemi, Anna-Liisa Levonen, Sohvi Hörkkö, Seppo Ylä-Herttuala

**Affiliations:** 1 Neurosurgery Research Group, Neuroscience program, Biomedicum Helsinki, Biomedicum, Helsinki, Finland; 2 Department of Biotechnology and Molecular Medicine, A.I.Virtanen-institute, University of Eastern Finland, Kuopio, Finland; 3 Department of Neurosurgery, Helsinki University Central Hospital, Helsinki, Finland; 4 Department of Medical Microbiology and Immunolgy, Institute of Diagnostics, University of Oulu and Nordlab Oulu, Oulu University Hospital, Oulu, Finland

**Keywords:** Intracranial aneurysm, Intima, Oxidized LDL, IgG, Acquired immunity, Inflammation

## Abstract

**Background:**

Rupture of a saccular intracranial aneurysm (sIA) causes an often fatal subarachnoid hemorrhage (SAH). Why some sIAs rupture remains unknown. Since sIA walls bear some histological similarities with early atherosclerotic lesions, we hypothesized that accumulation and oxidation of lipids might occur in the sIA wall and might associate with sIA wall degeneration. Tissue samples from sIA fundi (n = 54) were studied with histochemistry and a panel of previously characterized antibodies for epitopes of oxidized LDL (OxLDL). Plasma samples from sIA carriers (n = 125) were studied with ELISA and EIA for IgG and IgM -antibodies against a panel of OxLDL epitopes.

**Results:**

Lipid accumulation, foam cells, and oxidized lipids were found both in unruptured and ruptured sIA walls. Lipid accumulation associated with wall degeneration (P < 0.001), as did the expression of adipophilin, a marker of lipid ingestion by cells. Lipid accumulation associated also with loss of mural cells (P < 0.001), as did the accumulation of OxLDL (P < 0.001). Plasma IgG antibody titers against OxLDL or malondialdehyde modified LDL were higher in patients with unruptured sIAs than in patients with aneurysmal SAH (P ≤ 0.001). A trend but not statistically significant differences were found in plasma IgM antibodies against oxidized lipids.

**Conclusions:**

Accumulation of lipids and their oxidation in the sIA wall associates with the degeneration of the sIA wall. Acquired immunity against oxidized lipid epitopes may be protective of lipid associated sIA wall degeneration, but warrants further studies.

## Background

Subarachnoid hemorrhage (SAH) caused by saccular intracranial aneurysm (sIA) rupture is a rather common disease with a high fatality and morbidity. The incidence of aneurysmal SAH is 10–11 per 100 000 in North America and Europe, and twice as high in Finland and Japan [1]. Almost half of aneurysmal SAH patients die and half of survivors are left disabled [1,2]. Main risk factors for aneurysmal SAH are smoking, hypertension, female gender, familial background, and alcohol, caffeine, or cocaine abuse [2-4]. How these risk factors affect the sIA wall and trigger rupture, remains unknown.

Formation of an intracranial aneurysm does not always lead to eventual aneurysm rupture, and formation of a sIA seems to be a separate process from sIA rupture [5]. Knowledge of the pathobiology that leads to the degeneration of an existing sIA wall into a rupture-prone sIA, is necessary to identify sIAs at risk of rupture, and in order to develop novel therapies that would reduce the risk of sIA rupture.

Ruptured and unruptured sIA walls differ in histology [6-8]. Ruptured sIAs are characterized by a decellularized and degenerated matrix with an increased inflammatory cell infiltration, antibody accumulation, and activation of the complement system [6-8]. Histology of unruptured sIA walls often resembles the normal intima of an artery, or a hyperplastic intima that develops in hypertension or after mechanical injury [7,9]. What triggers loss of mural smooth muscle cells and increased inflammatory cell infiltration in the intima-like (or neointima-like) unruptured sIA wall is unknown.

Atherosclerosis is a chronic inflammatory disease of large and medium sized arteries [10-20]. Accumulation and oxidation of lipids in the intima is one of the main factors that induce and sustain chronic inflammation in atherosclerotic plaques [10-20]. Oxidized lipids can also directly induce cell death in the vascular wall [16-18]. In addition, oxidized LDL (OxLDL) in atherosclerotic intima activates the humoral immune system, which is mediated mainly by antibodies and the complement system [10-20]. Accumulation of antibodies and activation of the complement system in the sIA walls has been previously shown [8,21].

The presence of lipids and their oxidized epitopes have been shown in the sIA wall before [5,9,21], but association of lipids with wall degeneration has not been studied. We now investigated whether lipid accumulation and oxidation associates with sIA wall degeneration and rupture. In addition, we investigated whether the systemic immune response against oxidized lipid epitopes would associate with sIA rupture and subsequent SAH.

## Methods

### Tissue samples, plasma samples, and patient data

Tissue samples were collected intraoperatively from the fundi of 54 aneurysms after microsurgical clipping of the aneurysm neck. Samples were snap frozen in liquid nitrogen (n = 44), or fixed in 4% paraformaldehyde (PFA) for 6 hours (n = 10) and embedded in paraffin. As controls, two snap frozen samples of non-aneurysmatic MCA wall were obtained from ELANA bypass surgeries and two formalin fixed MCA bifurcations from autopsies. Blood samples were drawn with venipuncture on the 4th or 5th postoperative day from patients that underwent microsurgical clipping of unruptured or ruptured sIAs (n = 125). EDTA plasma was isolated by centrifugation. Medical records of the patients were reviewed for demographic data, medical history (smoking, hypertension, prior SAH, presence of other sIAs, family history of SAH, and history of cardiovascular or other major disease), and aneurysm size (Table 1 and Table 2). The study was approved by the Instutional Review Board and Ethical committee for the Departments of Neurology, Opthalmology, Otorhinolaryngology, and Neurosurgery of the Helsinki University Central Hospital.

**Table 1:** Patients demographics and clinical presentation of the aneurysms studied for lipid accumulation and oxidized lipids

**Variables**	**Bleeding status**		**P-value**
	***Unruptured***	***Ruptured***	
	**(n = 18)**	**(n = 36)**	
*A.Patients*			
Age (years)	55y. (42–70)	57y. (34–84)	0.773
Gender (females)	56% (10/18)	72% (26/36)	0.239
Patients with multiple sIAs (≥2)	33% (6/18)	36% (13/36)	1.000
History of prior SAH (from the studied sIA or another sIA)	20% (3/15)	97% (35/36) †	<0.001*
Familial background			0.174
Documented familial background (sIAs)	0% (0/18)	8% (3/36)	
Possible familial background #	11% (2/18)	11% (4/36)	
Family history unknown	39% (7/18)	58% (21/36)	
No familial background (verified cases)	50% (9/18)	22% (8/36)	
Smoking			0.129
Current smoker	50% (9/18)	39% (14/36)	
Ex-smoker	17% (3/18)	3% (1/36)	
Never smoked	17% (3/18)	17% (6/36)	
Status not known	17% (3/18)	42% (15/36)	
Hypertension	83% (15/18)	36% (13/36)	0.004*
*B.Aneurysms*			
Neck diameter (mm)	4.5 mm (3–7)	4 mm (1.5-8)	0.273
Width of fundus (mm)	6.0 mm (3–11)	7.0 mm (3–19)	0.624
Lenght of fundus (mm)	6.3 mm (3–11)	6.5 mm (2–27)	0.773

Median and range are given for continuous variables, proportions for categorical variables. Mann–Whitney U-test was used for continous and Fisher’s Exact test for categorical variables. P-values <0.05 are marked with *.

† One of the ruptured aneurysms presented in the operation with old thrombus and hemosiderin surrounding the aneurysm although the patient had not been diagnosed with SAH and had not had any symptomps suggesting SAH.

# When patients had multiple relatives that had been diagnosed with intracranial hemorrhage but the etiology of the hemorrhage was not clear.

**Table 2:** Demographics and clinical parameters of the patients studied for plasma antibodies against oxidized lipid epitopes

**Variables**	**Bleeding status**		**P-value**
	No SAH	Prior SAH	
	(n = 41)	(n = 84)	
A. Patients			
Age (years)	54y. (28–70)	55.5y. (11–84)	0.298
Gender (females)	71% (30/42)	71% (60/84)	1.00
Patients with multiple sIAs (≥2)	36% (15/42)	46% (39/84)	0.340
Patients with more than one known aneurysmal SAH	-	4% (3/84)	NA
Severity of the SAH (Hunt&Hess-grading)			NA
0 (no bleeding)	42	-	
1 (asymptomatic or mild headache / nuchal rigidity)	-	7% (6/84)	
2 (moderate headache / nuchal rigidity)	-	31% (26/84)	
3 (drowsiness/confusion, mild neurological deficit)	-	27% (23/84)	
4 (stupor, moderate-severe hemiparesis)	-	18% (15/84)	
5 (coma or decerebrate posturing)	-	8% (7/84)	
Hunt & Hess for the prior bleeding not known		7% (6/84)	
Known familial background	29% (12/42)	7% (6/84)	<0.001*
Smoking			<0.001 #
Current	50% (21/42)	42% (35/84)	
Ex-smoker	14% (6/42)	4% (3/84)	
Never smoked	36% (15/42)	21% (18/84)	
Status not known	0	32% (27/84)	
Hypertension	36% (15/42)	40% (34/84)	0.799

Median and range are given for continuous variables, proportions for categorical variables. Mann–Whitney U-test was used for continous and Fisher’s Exact test for categorical variables. *NA*, Not applicable.

* The percentage of unknown family histories is very high, especially in the PRIOR SAH group (76%, 64/84) making conclusions of the familial background unreliable.

#Although the difference of smoking habits between patients with unruptured sIAs and a history of aneurysmal SAH was highly significant, it is important to notice that in the SAH group the smoking history of 32% of patients was not unknown, which may significantly affect the result.

### Histochemistry and immunohistochemistry

Due to small size and limited availability of the tissue samples, all stainings were not performed from all samples, but instead subseries of samples were studied with different stainings.

For general morphology, paraffin and cryosections were stained with hematoxylin or hematoxylin-eosin stainings. To visualize lipids, cryosections were stained with Oil-Red-O followed by hematoxylin background staining for nuclei.

For the antibodies and dilutions used in immunohistochemistry, please see Table 3. Snap frozen tissue samples were cryosectioned at 4 um and fixed with −20°C acetone or 4% PFA, followed by endogenous peroxidase block with 3% hydrogen peroxide in methanol. Alternatively the peroxidase block was performed with 3% H202 in PBS between incubations with the secondary antibody and the avidin-biotion complex. The sections underwent serum block with 3% normal horse serum in PBS for monoclonal antibodies, or with 5% goat or rabbit serum for polyclonal antibodies. Following serum block the sections were incubated with the primary antibody either for 1 h at room temperature or overnight at +4°C, followed by PBS washes and incubation with a biotinylated secondary antibody (Vector, Burlingame, CA, USA). After subsequent PBS washes, the sections were incubated with either horseradish peroxidase or alkaline phosphatase conjugated avidin-biotin complex for 30 min – 60 min in room temperature. Peroxidase activity was visualized with DAB (Sigma-Aldrich, St. Louis, MO, USA and Zymed Laboratories Inc, San Fransisco, CA, USA) and alkaline phosphatase acivity with Vector Blue (Vector). Sections incubated with peroxidase conjugated primary antibodies underwent DAB incubation directly after primary antibody incubation and PBS washes. Mayer’s hematoxylin or Nuclear fast red were used as background stains for peroxidase or phosphate conjugated stainings. Stainings with the primary antibody omitted or replaced with an irrelevant antibody served as controls.

**Table 3:** Antibodies and dilutions used

**Antigens**	**Type**	**Clone / Name**	**Dilut.**	**Origin**	**Double stains with**
α-smooth muscle actin	mouse monoclonal	1A4	1:500	Sigma	HNE
CD45 (leukocyte antigen)	mouse monoclonal	2B11 + PD7/26	1:100	DAKO	Oil-Red-O
CD68 (macrophage)	mouse monoclonal	PG-M1	1:100	DAKO	15-LOX
15-Lipoxygenase	rabbit polyclonal	CheY	1:1000	(18)	CD68
*Native or oxidized LDL epitopes*					
ApoB-100	mouse monoclonal	MB47	1:500	(10)	-
Minimally modified LDL	mouse monoclonal	Ox4E6	1:200-1000	(13)	1A4, YE
Copper oxidized LDL	guinea-pig polyclonal	YE	1:500	(14)	Ox4E6
Hydroxynonenal (OxLDL)	guinea-pig polyclonal	HNE	1:1000	(11)	1A4, MDA-2
Malondialdehyde (OxLDL)	mouse monoclonal	MDA-2	1:50	(11)	EO6
Natural anti-OxLDL IgM from ApoE mice	mouse monoclonal	EO6	1:500-1000	(12)	1A4, MDA2

Sigma refers to Sigma-Aldrich Inc., St. Louis, MO. DAKO refers to DAKO A/S, Glostrup, Denmark. S-C refers to Santa-Cruz Biotechnology Inc, Santa-Cruz, CA, USA. For Anti-ApoB and oxidized LDL antibodies, the references were the antibodies were originally characterized are given in parentheses.

For double immunostaining, the sections underwent immunostaining with one antibody as described above, followed by a second serum block for 1 h or overnight, and immunostaining with a second primary antibody as described above, or with a fluorochrome conjugated secondary antibody (1:200, Alexa Fluor 488 green conjugated goat anti-mouse IgG, Molecular Probes Inc., Eugene, OR, USA) that was used to detect anti-alfa smooth muscle actin primary antibody (Table 3). Sections stained with fluorochrome conjugated secondary antibody were mounted with Vectashield containing DAPI (Vector).

Immunostainings were also performed on paraffin embedded tissue samples that were fixed for 6 h in room temperature with 4% PFA. Immunohistochemistry was performed as described for the frozen sections, but before the staining protocol the paraffin sections were deparaffinized in xylene and alcohol and underwent antigen retrieval in heated 0.01 M citrate buffer (pH 6).

### Histological analysis

The Oil-Red-O stained and copper oxidized LDL immunostained sections were classified according to the wall structure, as described previously (A: endothelialized wall with linearly organized SMC, B: thick myointimal hyperplasia-like wall with disorganized SMC, C: hypocellular wall with fresh or organizing thrombosis on the luminal surface, D: an extremely thin thrombosis-lined hypocellular wall. In this context, hypocellular refers to the low number of smooth muscle cells or myofibroblasts in the wall. The walls classified as hypocellular may have had significant inflammatory cell infiltration) [7].

**Figure 1:**
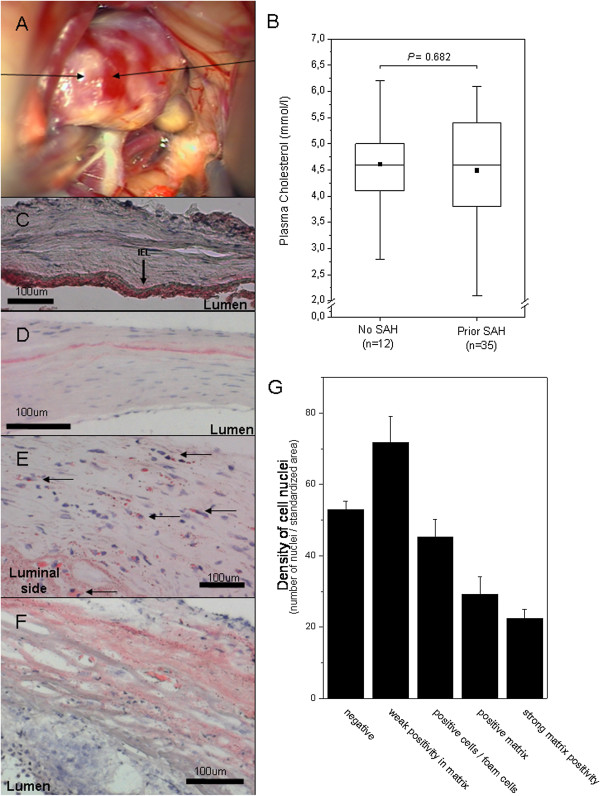
**Atherosclerotic changes, lipid accumulation, and degeneration of the aneurysm wall.** Intraoperative view of an MCA aneurysm with atherosclerotic changes **(A)**. Lipids accumulate to the aneurysm wall, despite plasma cholesterol levels that did not significantly differ from “normal” (**B**, bar graphs display mean and error bars SEM). In non-aneurysmal cerebral artery wall, lipid accumulation is mostly observed subendothelially on the luminal side of the internal elastic lamina (**C**, sample from a middle cerebral artery, polarized light). In most unruptured sIA walls that show features of mild intimal hyperplasia, lipid accumulation in Oil-Red-O stainings is limited to a matrix layer usually between the outer 1/3 and luminal 2/3 of the wall **(D)**. In aneurysms with more myointimal hyperplasia-like wall, intracellular lipid droplets and foam cells are seen **(E)**. In degenerated and decellularized aneurysm walls, Oil-Red-O staining is mostly extracellular and spread around the degenerated matrix **(F)**. Overall, in the Oil-Red-O stainings the accumulation of lipids was associated with loss of mural cells of the sIA wall **(G)**. The bar graph **G** displays mean values for density of nuclei (number of nuclei per a standardized surface area) and error bars present SEM.

**Figure 2:**
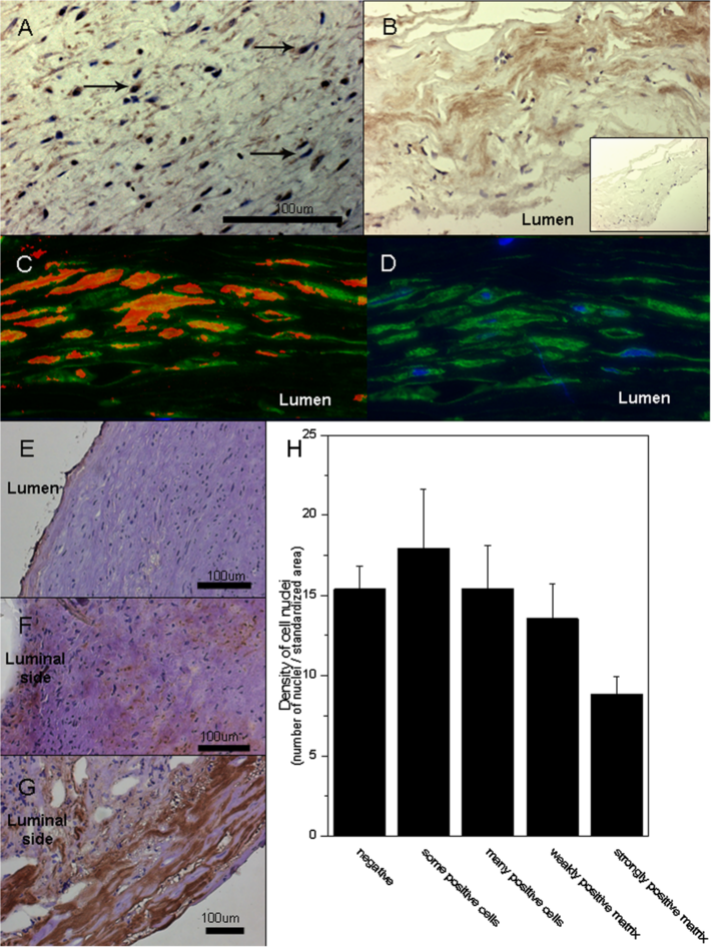
**Accumulation of LDL and oxidized lipids in the aneurysm wall.** Intracellular staining pattern for ApoB-100, the core protein of LDL, suggests uptake of cholesterol by cells in the aneurysm wall **(A)**. Staining for ApoB-100 is also observed in the aneurysm wall matrix (**B**, negative control in the insert). Double stainings **(C)** for hydroxynonenal (red) and alfa-smooth muscle actin (green) demonstrate accumulation of oxidized lipids in the smooth muscle cells of the aneurysm wall (**D**, negative control with only alfa-smooth muscle actin in green and nuclei in blue). The accumulation of oxidized LDL (as detected by immunostaining with an antibody against copper oxidized LDL, brown) associates with degeneration of the aneurysm wall (microphotographs: **E-G**, association with loss of mural cells **H**). The bar graph H displays mean values for density of nuclei (number of nuclei per a standardized surface area) and error bars present SEM.

Oil-Red-O stainings and immunostainings for copper oxidized LDL were imaged witht Zeiss Axiovision light microscope (Carl Zeiss GmbH, Germany). Consecutive serial microphotographs were taken with 10x magnification covering total surface area of the stained sections excluding luminal thrombus. From these microphotographs, the number of hematoxylin stained nuclei was counted with ImageJ (NIH software) from areas with and without lipid accumulation (Oil-Red-O staining) separately (n = 148 from 38 aneurysms), as well as from areas with and without oxidized LDL (immunostaining with YE antibody against copper oxidized LDL) separately (n = 101 from 15 aneurysms). Because the lipid accumulation varied a lot from a sample to another, and because areas with or without lipid accumulation were not of similar size within a section or sample, the number of counted nuclei was divided with the surface area of the region of interest from which the number of nuclei was counted from. This operation resulted to a variable describing the density of nuclei in wall areas with or without lipid accumulation. In addition, positive areas in Oil-Red-O and copper oxidized LDL stainings were scored for staining pattern according to the localization of the positive signal (intracellular or in the extracellular matrix) and according to signal intensity (few or many positive cells; weak, moderate, strong staining of the matrix, please see X-axis on Figure 1G for the scoring of Oil-Red-O stainings and X-axis on Figure 2H for the scoring of copper oxidized LDL immunostainings). Since multiple measurements were obtained from each sIA, Friedman test for non-parametric testing of related samples between multiple groups was used for statistical comparison of nuclear densities and staining patterns in Oil-Red-O or copper oxidized LDL immunostainings.

In addition to the multiple measurement from different sections of each individual sample described above, the ratio of positively stained surface area and total surface area were measured using Image J (NIH software) from reconstruction images made with Photoshop (Adobe Inc.) from consecutive microphotographs taken from the Oil-Red-O stainings and from immunostainings against copper oxidized LDL (YE antibody). Furthermore, for statistical comparison with clinical parameters, an average density of nuclei was calculated for the Oil-Red-O positive or negative and copper oxidized LDL positive and negative areas of each sIA sample from the multiple measurements performed from multiple sections of each sample.

Immunostainings for adipophilin, ApoB100, and oxidized lipid epitopes were scored as positive or negative and according to the localization of immunopositivity as follows: i) in the matrix, ii) in mural cells, or iii) in the thrombus. Microphotographs were taken with an Olympus AX70 (Olympus Optical, Japan) light and epifluorescent microscope using microscope mounted digital camera and analySIS (SoftImagingSystem GmbH, Germany) software. Overlay images and figure panels were created with Image J (NIH software) and Adobe Photoshop 8.0 software (Adobe Systems Inc., San Jose, CA, USA).

### Plasma lipid levels

Cholesterol and triglycerides levels were measured from plasma samples using standardized enzymatic methods at Kuopio University Hospital Central Laboratory.

### ELISA and EIA detection of anti-OxLDL antibodies in plasma samples

Titers of IgG against a modified ApoB-100 peptide (p244 sequence corresponding to the amino acids 3144–3163 of ApoB-100) were measured with a commerically available ELISA kit (Ark Therapeutics Group Inc., London, UK). Plasma samples were incubated in plates coated with the modified peptide. Following subsequent washes, incubation with peroxidase conjugated anti- human IgG reagent and subsequent new round of washes, the plates were incubated with peroxidase substrate (H202) and TMB chromogen for 30 min. Color development was stopped with 0.5 mol/L H2SO4 , and absorbances were measured at 450 nm with a Multiskan microplate reader (Thermo LabSystems, Waltham, MA, USA).

Titers of IgG against copper oxidized and native LDL were first measured as described previously by Närvänen et al. [22]. In brief, plasma samples were incubated in 96 well plates coated with either native and copper-oxidized LDL as described previously [22]. After subsequent washes, bound IgG was detected with a peroxidase conjugated anti-human IgG reagent and TMB chromogen as described [22], using the Multiskan microplate reader. Circulating IgG antibodies against “native” and copper oxidized LDL were measured in three groups: first in 24 patients with SAH and 11 patients with unruptured sIAs, then in other 21 patients with SAH and 11 patients with unruptured sIAs, and finally in a series of 65 patients with SAH and 32 patients with unruptured sIAs, which included the patients in both prior groups. All measurements in both types of ELISA were performed as duplicates in each plates. To control for variance between the plates, 34% of the samples were tested in two or three plates.

Plasma IgG and IgM antibody levels to copper oxidized LDL and malondialdehyde modified LDL, were also determined with chemiluminescent immunoassay as previously described [23]. Copper oxidation and malondialdehyde (MDA) modification of LDL were prepared as described [24]. Antigens were immobilized on white 96-well microtiter plates at 5 μg/ml in PBS containing 0.27 mM EDTA overnight at +4°C. Plasma samples were diluted 1:500 and incubated for 1 h at room temperature. The amount of plasma antibodies bound was measured with alkaline phosphatase labeled anti-human-IgG and anti-human-IgM antibodies (Sigma-Aldrich). LumiPhos 530 (Lumigen Inc., Southfield, MI, USA) was used as substrate and the chemiluminescence was measured with Wallac Victor3 multilabel counter (Perkin Elmer, Waltham, MA, USA). The results were expressed as relative light units measured in 100 ms (RLU/100 ms).

### Statistics

Proportions, medians, and range were calculated for categorical and continuous variables and compared with 2-sided Fisher’s exact test, Friedman test, Mann–Whitney U-test, or Spearman’s correlation test as approriate. Statistics were calculated using SPSS 17.0 statistical software (Apache Software Foundation). Alpha-level was 0.05. Graphs were drawn with OriginPro 8.6 software (OriginLab Corporation, Northhampton, MA, USA).

## Results

### Accumulation of neutral lipids in intracranial aneurysm wall

Lipid accumulation in Oil-Red-O stainings was detected in all studied snap frozen sIA walls (n = 38, 13 unruptured). Lipid accumulation in sIA walls was observed despite normal plasma cholesterol and triglyseride levels (median chol 4.0 mmol/l, range 2.1-4.7 and trigly 1.1 mmol/l, range 0.6-3.2, n = 10 patients included in the histological series, please see Figure 1B for plasma cholesterol levels in patients studied for circulating antibodies reactive against oxidized lipids). In samples obtained from two non-aneurysmal middle cerebral artery walls, in one accumulation of lipid was observed in the subendothelium but not in the media (Figure 1C), and in the other throughout the wall.

In sIA walls that were abundant in mural cells, lipids were found in the matrix in a rim between the outer third and the inner two thirds of the wall (Figure 1D), but mostly intracellularly (foam cells) (Figure 1E). In sIA walls that had less mural cells and more degenerated matrix, the accumulated lipid was found mostly in the matrix and throughout the wall (Figure 1F). Although lipid accumulation was found in both unruptured and ruptured sIAs, the pattern of lipid accumulation (scored as described on the X-axis of Figure 1G) was associated with wall degeneration (scored from A-D as described, P < 0.001, Fisher’s exact in both unruptured and ruptured sIAs respectively) and loss of mural smooth muscle cells (number of nuclei per standardized surface area, P < 0.001, Friedman test, Figure 1G). The more lipid in the matrix, the less that region had cells.

### Ingestion of lipids by mural cells

**Figure 3:**
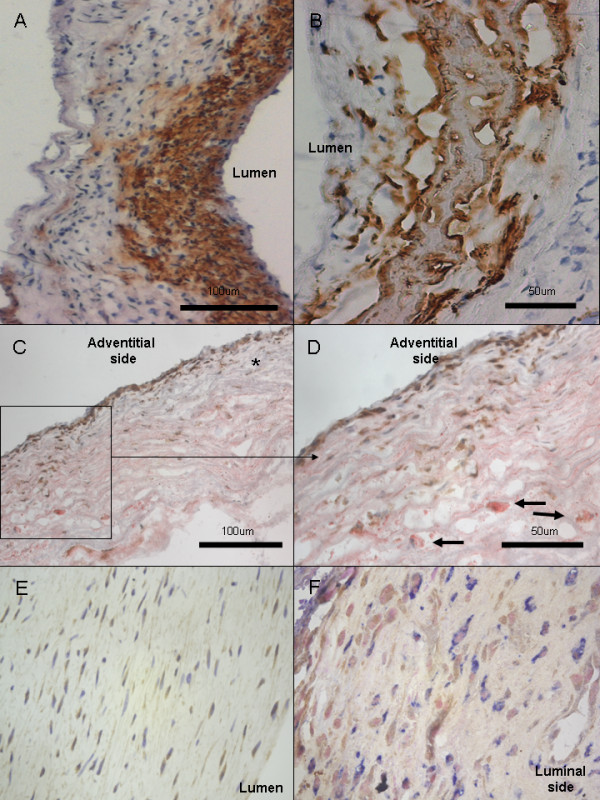
**Ingestion of lipids by mural cells and inflammatory cells.** Adipophilin was expressed in the mural cells of intimal hyperplasia or myointima -like sIA walls **(A)**. In degenerated sIA walls, adipophilin staining does not only stain cells but mostly extracellular debris **(B)**. CD45 and Oil-Red-O double stainings showed presence of inflammatory cells in wall areas abundant in extracellular lipid (**C-D**, CD45 in brown with DAB, Oil-Red-O and hematoxylin background stain). CD45 positive cells were also found in areas without lipid accumulation (marked with * in **C**). Most foam cells observed were CD45 negative (marked with arrows in **D**) and therefore not leukocytes. Expression of 15-lipoxygenase, a known intracellular oxidant of lipids (18), was found in aneurysm wall cells (**E**, 15-lipoxygenase in brown with DAB and hematoxylin background stain, x40 magnification). Double stainings with CD68 (**F**, 15-lipoxygenase in brown with DAB and CD68 in blue with AFOS, nuclear fast red background stain) demonstrated that most cells expressing 15-lipoxygenase were not CD68+ macrophages **(F)**, although some double positive cells were also found (data not shown).

Expression of adipofilin, a protein induced by OxLDL ingestion on smooth muscle cells and macrophages [25], was observed in the sIA wall (6 ruptured and 5 unruptured sIAs studied). Adipofilin positive cells were found in areas of cellularized, intimal hyperplasia-like wall (Figure 3A), whereas in decellularized and degenerated walls adipofilin staining localized to the debris in the extracellular matrix (Figure 3B).

### Accumulation of LDL particles in the aneurysm wall

Immunostaining for ApoB100 (the core protein of LDL) was observed in sIA walls (10/10, 5 unruptured Figure 2A-B). In many sIA walls, ApoB100 had an intracellular staining pattern concordant with ingestion of LDL particles by mural cells (Figure 2A). ApoB100 was, however, found also in the extracellular matrix in some samples (Figure 2B).

The sIA walls underwent immunostainings with a panel of previously characterized antibodies generated against oxidized LDL epitopes (Table 3). Immunopositivity for epitopes of oxidized LDL were found in all stained samples (23/23, 5 unruptured, data not shown). Antibodies against copper oxidized LDL colocalized with immunopositivity for minimally modified LDL (Ox4E6 antibody) that stained a larger area of the wall. Immunopositivity for both malondialdehyde and hydroxynonenal (neoepitopes formed by oxidation of LDL) was also found in sIA walls, and colocalized in double stainings with natural mouse IgM antibodies reactive to oxidized LDL (EO6). Double stainings with alfa smooth muscle actin and hydroxynonenal showed ingestion of oxidized lipids by mural smooth muscle cells (Figure 2C-D).

Similarly to Oil-Red-O stainings, staining pattern (scored as described on the X-axis of Figure 2H) for the copper oxidized LDL (n = 15, 2 unruptured) was associated with loss mural smooth muscle cells (number of nuclei per standardized surface area, P < 0.001, Friedman test, Figure 2H). In walls abundant in cells, oxidized LDL was found more intracellularly, although some matrix staining was also observed (Figure 2E-F). In the degenerated walls with loss of mural cells, staining for OxLDL was mostly located to the matrix (Figure 2G). The extent of copper oxidized LDL immunostaining (measured as positive surface area / total surface area of the histological section) correlated (rho = 0.71, P = 0.003, Spearman rank) with the extent of lipid accumulation in Oil-Red-O stainings (measured as positive surface area / total surface area of the histological section ). In the two autopsy samples from non-aneurysmal middle cerebral artery bifurcation, immunopositivity for copper oxidized LDL was found in pads of intimal hyperplasia near the bifurcation (data not shown).

### Natural antibodies (IgM) do not associate with aneurysm rupture

**Figure 4:**
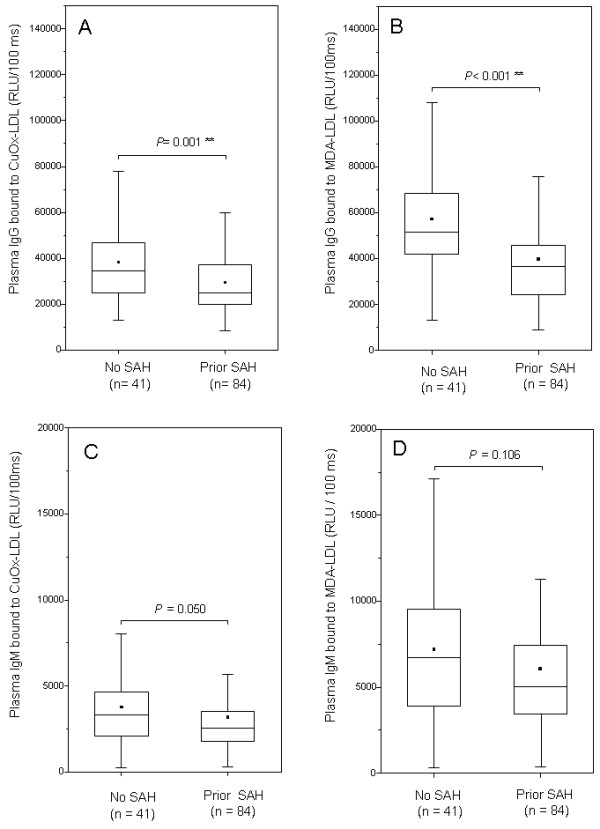
**Antibodies against oxidized lipids in the plasma.** Titers of plasma IgG reactive against oxidized LDL were significantly higher in patients with intracranial aneurysms but no history of SAH **(A)**, as were also the titers of plasma IgG against malondialdehyde **(B)**. Titers for plasma IgM reactive against oxidized LDL **(C)** or malondialdehyde **(D)** did not statistically differ among patients with a history of SAH or no history of SAH, although there was a trend towards similar findings as with IgG titers. Box plots represent the median value (horizontal line) and the 25^th^ and 75^th^ percentiles (box edges). Range is given with error bars and the small black box displays mean values.

Plasma IgM antibodies against copper oxidized LDL or malondialdehyde modified LDL, did not statistically significantly differ between patients with unruptured sIAs or SAH, although there was a trend for lower levels in patients with a history of SAH (Figure 4C-D).

### Acquired humoral immunity (IgG) against oxidized lipid epitopes associates with aneurysm rupture

Plasma IgG antibodies against a modified peptide of the ApoB100 protein were found in 40/92 (43%) of the sIA patients, and had an increasing trend in SAH patients (33% vs. 50%) but failed to reach statistical significance (n = 92). Inspired by the trend, IgG antibodies against oxidized or native LDL were measured as described by Närvänen et al. [22] and by Karvonen et al. [23].

Contrary to the antibodies against modified ApoB100, levels of OxLDL or native LDL reactive IgGs measured as described by Närvänen et al. [22] were significantly higher in patients with unruptured sIAs when compared to SAH patients (n = 97 of which 32 unruptured, median for OxLDL reactive antibodies 0.59 vs 0.47, P = 0.021 Mann–Whitney U, for unruptured sIAs and SAH patients respectively and median for native LDL reactive antibodies 0.43 vs 0.35 for unruptured sIAs vs. SAH patients, P = 0.008 Mann–Whitney U). Their ratio did not significantly differ. To confirm the findings, plasma IgG levels were also measured by chemiluminescent immunoassay against copper oxidized LDL and malondialdehyde modified LDL. Levels of IgG antibodies against these epitopes were significantly higher in patients with unruptured sIAs when compared to SAH patients (n = 125, P = 0.001 and <0.001, Mann–Whitney U, Figure 4A-B).

### Associations of oxidized lipid reactive IgG levels and known clinical risk factors for SAH

Patient demographic data and aneurysm characteristics are shown in Table 2.

The association of IgG antibodies against OxLDL or malondialdehyde modified LDL with SAH was clear in females (median RLU for antibodies against OxLDL: 34 276 in unruptured sIA patients vs 25 127 in SAH patients, P < 0.006, Mann–Whitney U and median RLU for malondialdehyde modified LDL: 48 100 in unruptured sIA patients vs 34 490 in SAH patients, P < 0.001, Mann–Whitney U) but only a statistical trend in males (P = 0.067, 0.112) respectively, Mann–Whitney U). In female patients, age correlated inversily with malondialdehyde modified LDL reactive IgG antibodies (rho = −0.234, P = 0.027, Spearman rank).

The association of IgG antibodies against OxLDL or malondialdehyde modified LDL with SAH was significant in patients with single sIAs (median RLU for antibodies against OxLDL: 34 727 in unruptured sIA patients vs 22 373 in SAH patients, P = 0.001, Mann–Whitney U and median RLU for malondialdehyde modified LDL: 51 204 in unruptured sIA patients vs 31 821 in SAH patients, P < 0.001, Mann–Whitney U), but in patients with multiple sIAs the association of IgG against OxLDL was only a trend (P = 0.220) and only the IgG against malondialdehyde modified LDL was significantly different in patients with prior SAH vs. unruptured sIAs (median RLUs: 39 410 vs. 51 417, P = 0.030, Mann–Whitney U).

Smoking was slightly more prevalent in patients with unruptured sIAs (50% vs 42% in SAH patients). However, the smoking status of 32% of the SAH patients was not obtained, making it possible that smoking was much more prevalent than reported among SAH patients (Table 2). In SAH patients, confirmed current ex-smokers (n = 35) had higher levels of IgG against malondialdehyde modified LDL than confirmed non smokers (n = 18) (P = 0.017, Mann–Whitney U). In patients with unruptured sIA smoking did not, however, associate with IgG titers.

Hypertension was not clearly associated with SAH in this sample of patients (40% vs 36%, Table 2). Neither was hypertension associated with the titers of oxidized lipid reactive IgG or IgM titers in patients with SAH or unruptured sIAs, in female or male sIA patients, nor in patients with single or multiple sIAs.

Diagnosed cardiovascular diseases were found only in a few cases. Total cholesterol and triglyseride levels were measured from the plasma of 35 patients with SAH and 12 patients with unruptured sIAs, included in the serology studies. No difference was found in the plasma levels of total cholesterol or triglyserides between the groups (Median plasma cholesterol: 4.6 mmol/l, range 2.8-6.2 mmol/l for unruptured sIA patients and 4.6 mmol/l, range 2.1-6.1 mmol/l for patients with SAH history; median plasma triglycerides: 1.6 mmol/l, range 0.6-5.0 mmol/l for unruptured sIA patients and 1.0 mmol/l, range 0.5-5.7 mmol/l for patients with SAH history, Figure 1B). Furthermore, neither cholesterol nor triglyseride levels were unusually high in either group. No correlation was found between either between the plasma cholesterol or triglyseride levels and IgG or IgM antibodies against oxidized LDL epitopes. Smoking and hypertension did not either associate with plasma lipid profile in this patient population.

## Discussion

We showed that lipids accumulate in sIA walls in all sIA patients despite normal lipid levels, and that those lipids are ingested by cells in the sIA wall, become oxidized and turn in the sIA wall into immunogenic and cytotoxic neoepitopes. Furthermore, we showed for the first time that this lipid accumulation is associated with the degeneration and tendency of the sIA wall to rupture. This suggests that lipid accumulation and oxidization has a role in the degeneration of the aneurysm wall towards a rupture-prone wall. We also observed higher levels of IgG antibodies against oxidized lipids in patients with unruptured sIAs than in patients with aneurysmal SAH, suggesting that immune response against oxidized lipids may regulate lipid and oxidized neoepitope induced sIA wall degeneration.

### Age and accumulation of lipids in unruptured and ruptured intracranial aneurysms

Visible atherosclerotic changes were found during operation in the parent arteries in 37% of patients with unruptured sIAs included in this study, and in 37% of patients with SAH and included in this study. In histological studies, however, accumulation of lipids was found in all studied sIA walls. Accumulation of lipids was found also in the 4 “normal” MCAs that were studied. Taken together, this suggests that accumulation of lipids in the cerebral vasculature is a somewhat common phenomenon, probably related to aging. What differs between unruptured and ruptured sIA walls is the pattern of lipid accumulation, which associates with loss of cells and degeneration of the wall.

The mechanisms how LDL and other lipids accumulate in the sIA wall remain unknown. Exposure of the endothelium to increased hemodynamic stress, e.g. in hypertension, predisposes to disturbances in the lipid metabolism of the arterial wall and subsequent intimal accumulation of lipids that become chemically modified [16]. Aneurysm walls are exposed to non-physiologic hemodynamic shear stress [26], suggesting that altered lipid metabolism of the sIA wall endothelia or complete or partial loss of endothelia and its barrier function, might be a mechanism that leads to accumulation of lipids to the sIA wall.

### Oxidized lipids accumulate in the mural smooth muscle cells and in the matrix of the sIA wall

Our results show that the lipids that have accumulated to the aneurysm wall become ingested by mural smooth muscle cells and infiltrating macrophages, both of which eventually may turn into lipid ladden foam cells. In these foam cells, the ingested lipids are known to be enzymatically modified, by e.g. 15-lipoxygenase (15-LOX) [27], which we found expressed in the sIA wall cells (Figure 3E-F). It seems therefore likely, that the lipids in the sIA wall become oxidized and modified in the cells that have ingested them, similarly to what happens in atherosclerotic lesions [27]. It is, however, also possible that some of the lipids are oxidized extracellularly by oxidants released from luminal thrombus, infiltrating inflammatory cells, or mural cells that undergo necrotic cell death [5,28].

### The effect of oxidized lipids in the aneurysm wall

The oxidized epitopes that we found in the sIA wall matrix and in smooth muscle cells, are potent inducers of cell death in vascular smooth muscle cells [18]. We showed an association between accumulation of lipids or oxidized LDL and the loss of mural cells and degeneration of the sIA wall. This suggests that accumulation of lipids or oxidized lipids might be an inducer of cell death in the sIA wall.

Oxidized lipids induce chronic inflammation in the atherosclerotic intima [10-20]. As in atherosclerosis, infiltration of macrophages and T-cells is increased in ruptured sIA walls [5-7]. Although we cannot establish a causality link between accumulation of oxidized lipids in the sIA wall and sIA wall inflammation, our observations and prior knowledge of the effects of oxidized lipids in the arterial wall suggest that accumulation of oxidized lipids in the aneurysm wall may trigger or maintain the cellular and humoral inflammation observed in sIA walls. Moreover, the oxidized lipids that are ingested by phagocytosis in antigen presenting cells (macrophages) in the sIA wall, may trigger a systemic humoral inflammatory response against oxidized lipid epitopes. Inflammatory cells were observed in the sIA wall areas with lipid accumulation (Figure 3C-D), although most foam cells observed were CD45 negative (not leukocytes).

### Acquired humoral immunity against oxidized lipids - Protective of sIA wall degeneration and rupture?

Accumulation of oxidized lipids in the vasculature is known to trigger an immune response that leads to the formation of acquired antibodies against these oxidized lipids [12,13,15-17,20]. The plasma titers of these acquired oxidized lipid reactive antibodies correlate with the risk of clinical manifestations of atherosclerosis, although depending on the method used in the measurements, the correlation can be positive or negative [20,22-24,29,30]. Although the role that ox-LDL reactive antibodies have in atherosclerosis and lipid associated vascular diseases is not completely known, it has been shown in animal models that induction of acquired antibodies against oxidized lipids with immunization can reduce and even protect from the formation of atherosclerosis [15,20]. It seems that at least some of the antibodies against oxidized lipids are protective and may facilitate the clearance of oxidized lipids from the vascular wall [15,20].

In the patients we studied, lower levels ox-LDL reactive IgG antibodies were associated with a history of sIA rupture and SAH in patients with sIAs. Our results could suggest that patients with unruptured sIAs have acquired a stronger humoral immunity against oxidized lipids than patients in whom sIAs have ruptured and caused SAH. Since it is possible that consumption of IgG antibodies after SAH might have influenced the results, although no difference was found in IgM class antibodies reactive to oxidized lipids, our observation and interpretation should be confirmed with prospective serology studies on patients with unruptured sIAs.

### Serology as a clinical tool to predict rupture risk in sIAs?

Despite contradictory results, screening for anti-OxLDL antibodies has been suggested to be a useful diagnostic tool to detect patients at an increased risk of acute cardiovascular events [20,22-24,29,30]. The value of anti-OxLDL antibodies to detect patients at risk of hemorrhagic stroke has been investigated in one study [30], which found a negative result [30]. However, in that study two etiologically different forms of hemorrhagic stroke were grouped together (spontaneous intracerebral hemorrhage and aneurysmal hemorrhage) [30], which very likely influenced the outcome.

In order to use the titers of circulating antibodies reactive against oxidized lipids as a meaningful tool in the estimation of SAH risk in patients with unruptured sIAs, the results of this study should be replicated in separate, large prospectively collected patient cohorts and the possible associations with SAH risk factors investigated. Standardization of the methods will be of utmost importance in order to get consistent results in different cohorts.

## Conclusions

We show that lipids accumulate to the intracranial aneurysm wall, where they are ingested by mural cells and oxidized. We demonstrate that the accumulation of lipids and oxidized lipids in the aneurysm wall is associated with loss of mural cells, degeneration of the wall, and eventual aneurysm rupture. In addition, we show differences in the systemic immune response against oxidized lipids in patients with ruptured and unruptured aneurysms.

## Competing interests

Drs. Tommi Heikura and Seppo Ylä-Herttuala have had employee and consultant relations with Ark Therapeutics Inc. that supplied ELISA kits used in the detection of circulating antibodies reactive against modified ApoB-100. Other authors declare that they have no competing interests.

## Authors’ contributions

JF: Drafted the original hypothesis, collected the samples and clinical data, performed most of the immunohistochemistry and ELISA lab work, analysed the data, interpreted the results and wrote the manuscript. Contributed also to acquisition of funding. RT: Contributed in the drafting of the original hypothesis, helped with the immunohistochemistry, contributed to data analysis and interpretation, as well as writing of the manuscript. TH: Performed part of the ELISA lab work and contributed to the data analysis and interpretation of the data. SS: Performed a significant part of the histological analysis and contributed to the data analysis and interpretation of the data. MN: Contributed to sample collection, acquisition of funding, interpretation of the data and writing of the manuscript. JH: Contributed to sample collection, acquisition of funding, interpretation of the data and writing of the manuscript. ALL: Contributed to the drafting of the original hypothesis, helped with the immunohistochemistry, contributed to data analysis and interpretation, as well as writing of the manuscript. SH: Contributed to the drafting of the original hypothesis, performed the EIA analysis, contributed to data analysis and interpretation, as well as writing of the manuscript. SYH: Contributed to the drafting of the original hypothesis, acquisition of funding, analysis and interpretation of the data, and writing of the manuscript. All authors read and approved the final manuscript.
